# NAVIGATING LIFE’S PHASES: CONTROL PERCEPTIONS AND GOAL ORIENTATION IN YOUNGER AND OLDER AGE GROUPS

**DOI:** 10.1093/geroni/igad104.3487

**Published:** 2023-12-21

**Authors:** Ryan Muskin, Eric Allard, Sachin Suri

**Affiliations:** Cleveland State University, Cleveland, Ohio, United States; Cleveland State University, Cleveland, Ohio, United States; Cleveland State University, Cleveland, Ohio, United States

## Abstract

This study examines age-related differences in the perceptions of control and goal orientation among younger adults (ages 18-35) and older adults (aged 60+). A cohort of 57 younger adults and 51 older adults were exposed to a series of emotionally evocative videos portraying diverse scenarios involving varying levels of control and distinct growth or preservation goals. Participants evaluated perceived control, growth orientation, and preservation orientation using 10-point Likert scales. Our findings reveal significant disparities between the two age groups. Older adults exhibited diminished perceptions of control across all videos (p < .001) compared to their younger counterparts. Conversely, younger adults consistently displayed a heightened sense of growth orientation across the videos (p < .001), without significant age-based variations in perceptions of preservation goals. These outcomes contribute to our comprehension of developmental trajectories in perception and relevance appraisal, along with emotion evaluation. Our hypothesis posits that the disparity in control perceptions stems from age-related losses such as diminished cognitive and physical capabilities, as well as experiences like bereavements. In contrast, the robust growth, competition, and development characteristic of younger adulthood may foster the tendency to perceive the world through these lenses. By shedding light on the intricate interplay between age, control perceptions, and goal orientations, this study advances our understanding of cognitive and emotional dynamics across the lifespan. The implications are far-reaching, offering insights into potential interventions for age-related emotional well-being and adaptive coping strategies.

